# Soil organic carbon and ecosystem multifunctionality are enhanced by subsoiling in fluvo-aquic soil of North China Plain

**DOI:** 10.3389/fpls.2025.1559653

**Published:** 2025-04-16

**Authors:** Mengru Wang, Renzhuo Wang, Guiying Jiang, Yueying Li, Chaolin Liu, Jin Yang, Xiaolei Jie, Fengmin Shen, Fang Liu, Shiliang Liu

**Affiliations:** Key Laboratory of Arable Land Quality Conservation in the Huanghuaihai Plain, Ministry of Agriculture and Rural Affairs, College of Resources and Environment, Henan Agricultural University, Zhengzhou, China

**Keywords:** subsoiling, fluvo-aquic soil, organic carbon component, soil enzyme activity, soil ecosystem multifunctionality

## Abstract

This study investigated the impact of various tillage modes on soil carbon (C) components, crop yield, enzyme activity, and ecosystem multifunctionality (EMF) in the North China Plain (NCP), aiming to determine the most effective tillage practice for C sequestration in the region. Field experiments were conducted from 2016 using a split-plot design that included rotary tillage (RT) and deep tillage (DT) during the wheat season and no-tillage (NT), subsoiling in-row (SIR), and subsoiling inter-row (SBR) during the maize season. Related tillage modes based on the total amount of straw returned. Soil bulk density (BD), soil C components, soil organic carbon (SOC) storage, enzyme activities, soil quality index (SQI), EMF, and wheat yield were measured and analyzed. Compared to rotary tillage-no-tillage (RT-NT), the BD of the 0–40 cm soil layer decreased under the other treatments during 2018–2019. The C component content decreased with soil depth across all treatments. Treatments incorporating subsoiling during the maize season led to higher SOC, labile organic carbon (LOC), non-LOC, and microbial biomass carbon (MBC) in the 20–40 cm soil layer. DT-SBR and DT-SIR increased SOC storage. Enzyme activities were highest in the 0–20 cm soil layer under RT-SBR and RT-SIR, while in the 20–40 cm soil layer, enzyme activity peaked under DT-SBR and DT-SIR. The highest SQI value in the 0–20 cm layer was observed under RT-SBR and RT-SIR in both years. Meanwhile, the highest EMF values were under DT-SIR and DT-SBR in the 30–40 cm layer in 2018, ranged from −0.79 to −0.08. Key factors influencing EMF included MBC, LOC, SOC, and dissolved organic carbon (DOC), with EMF showing a strong positive correlation with SQI. Subsoiling during the maize season enhanced wheat yield, with the highest values for RT and DT being 6697 and 6587 kg ha^-1^, respectively. In conclusion, DT during the wheat season and subsoiling during the maize season promoted the transformation of SOC, enhanced yield, enzyme activity, SQI, and EMF. These benefits contributed to greater C sequestration in deeper soil layers, offering a sustainable approach to soil management in the fluvo-aquic soils of the NPC.

## 1 Introduction

Soil organic carbon (SOC), the largest terrestrial carbon pool, plays a crucial role in global carbon (C) cycling (Rocci et al., 2021; Meng et al., 2024). SOC is generally divided into labile and stabilized organic C fractions. Labile fractions, highly sensitive to plant and microbial activity, provide an accurate and timely reflection of short-term SOC fluctuations. In contract, stabilized fractions resist decomposition, contributing to long-term SOC sequestration (Lal, 2004; Haynes, 2005; Benbi et al., 2015; Chen et al., 2016). Both natural processes and human activities influence soil C components (He et al., 2021; Wooliver and Jagadamma, 2023). In agricultural ecosystems, tillage is an important management practice affecting SOC turnover by directly disturbing soil structure, which in turn influences the soil’s response to climate change. While different tillage methods have different effects on SOC, no-tillage with straw incorporation generally sequesters more SOC than conventional tillage, as surface crop residues reduce organic matter mineralization (Bai et al., 2019; Sun et al., 2020). However, long-term no-tillage causes soil compaction, limiting roots growth and reducing root-derived C input into deeper soil layers. This results in SOC accumulation at the surface while restricting sequestration in subsoil layers (Blanco-Canqui and Ruis, 2018). Additionally, increased soil bulk density (BD) due to no-tillage has been found to lower rice yields (Zhang et al., 2023b). Studies have shown that SOC content in the 0–10, 10–20, and 20–40 cm soil layers increased by 42.7%, 19.2%, and 73.3%, respectively, under rotary tillage with straw incorporation compared to initial value (Yu et al., 2024). However, prolonged rotary tillage with straw returning at a consistent depth can lead to soil compaction and a shallow tillage layer, concentrating organic materials in surface soil and limiting their downward movement, thereby creating a pronounced stratification of SOC (Raper and Bergtold, 2007; Shah et al., 2017). A meta-analysis found that deep tillage enhanced SOC stock in the 10–40 cm layer while have no effect on the SOC in the 0–10 cm layer (Wang et al., 2023). Additionally, combining rotary and deep tillage had been shown to enhance crop yields by improving soil physical structure and facilitating SOC accumulation in deeper layers (Li et al., 2024). Subsoiling, a tillage technique that loosens compacted soil, promotes root penetration into subsoil layers while preserving soil structure and minimizing SOC loss through mineralization (Feng et al., 2020). This method has been widely adopted, with extensive research highlighting its benefits for soil physical and chemical properties. Subsoiling enhances soil quality and SOC sequestration (Xu et al., 2019; Zhang et al., 2023). Long-term field experiments show that transitioning from rotary tillage to subsoiling increases SOC accumulation, resulting in a 22% increase in SOC content and a 17% increase in C storage compared to conventional tillage (Tian et al., 2016). Tian et al. (2016) further reported that subsoiling during wheat season significantly increased total SOC content in the 0–30 cm soil layer, despite decreases in light fraction organic carbon, soil microbial biomass carbon (MBC), and dissolved organic carbon (DOC). Furthermore, Nie et al. (2015) showed that subsoiling significantly increased the annual yield of wheat and maize. Overall, implementing an appropriate tillage system can enhance SOC storage, improve soil quality, and support sustainable crop production.

Soil microorganisms drive SOC transformation, with their extracellular enzymes playing a direct role in soil biochemical processes (Wang et al., 2022; Huber et al., 2024). These enzymes, which regulate organic matter decomposition, are highly sensitive to agricultural practices. Conservation tillage has been shown to enhance enzyme activity, particularly hydrolases and oxidases, compared to conventional tillage (Wang et al., 2022). Key enzymes such as cellobiohydrolase, β-glucosidase, β-xylosidase, β-N-acetylglucosamines, and alkaline phosphatase showed higher activities under different tillage treatments. By altering soil physical and chemical properties, tillage practices influence enzyme dynamics, thereby affecting SOC content and transformation (Hou et al., 2016; Ren et al., 2024). Despite extensive research on surface soil enzyme activity, the effects of different tillage practices on enzyme activity in deeper soil layers remain underexplored.

Ecosystem functions, which include biological and geochemical processes, are closely linked to ecosystem services and their economic value (de Groot et al., 2002; Scherzinger et al., 2024). In agricultural systems, tillage practices shape the microenvironment for soil microorganisms and crops, impacting microbe-mediated biochemical reactions and overall ecosystem functioning (He et al., 2021; Mihelič et al., 2024). To develop and implement sustainable practices, it is essential to evaluate soil characteristics under different management systems (Jia et al., 2022). The ecosystem multifunctionality (EMF) index, derived from multiple enzyme activity measurements, is widely used to assess soil function and biodiversity (Luo et al., 2018). The soil quality index (SQI) integrates physicochemical diagnostics and biogeochemical cycling metrics to holistically evaluate soil’s capacity to sustain bioproductivity, regulate ecosystem integrity, and buffer environmental stressors (Zhu et al., 2025). However, few studies have investigated how the EMF and SQI index responds to tillage management and soil depth in an agroecosystem.

The North China Plain is a crucial agricultural region in China, is characterized by fluvo-aquic soil, which covers approximately ~33% of this area (Xi, 1998; Ju and Zhang, 2017; Sun et al., 2023). Common tillage practices include rotary tillage before winter wheat sowing and no-tillage during summer maize cultivation in this area (Kong et al., 2012). However, these practices have led to a shallower topsoil layer and an elevated plow bottom, which enrich surface soil nutrients but restrict root growth and hinder SOC turnover (Liu et al., 2010; Zhai et al., 2017). Previous research on subsoiling during the maize season has primarily focused on single-cropping or double cropping during wheat seasons, with limited studies examining subsoiling during the maize season in wheat-maize rotations. Additionally, most studies have overlooked deeper soil layers, which may experience degradation and restricted crop yields due to current tillage practices. Therefore, further research is needed on assess the impact on SOC components and EMF at deeper soil layers. This study aims to investigate changes in SOC content, SOC storage, soil enzyme activity and EMF across different soil layers under various tillage combinations. Specifically, it examines the effects of rotary and deep tillage during the wheat season, combined with subsoiling or no-tillage during the maize season. The aim is to establish a theoretical basis for selecting appropriate tillage methods in wheat-maize double-cropping areas in the North China Plain. The hypotheses are as follows: (i) deep tillage with subsoiling will decrease soil BD via intensifying the disturbance to the soil and enhancing soil porosity; (ii) deep tillage with subsoiling will increase the content of different SOC components, organic carbon storage, and yield by bring straw into soil and promoting nutrition transformation; (iii) deep tillage with subsoiling will improve SQI and EMF. It may provide an effective approach to sustainable soil management in fluvo-aquic soil of the North China Plain.

## 2 Materials and methods

### 2.1 Site description

The experiment was carried out in Yuanyang, Henan province (34°47′N, 113°40′E) from 2016. The site has a warm temperate continental monsoon climate, with an annual mean temperature of 14.5°C, annual mean precipitation of 616 mm, and annual evaporation of 1461 mm. The soil type is fluvo-aquic soil, with a double-cropping system of winter wheat (*Triticum aestivum* L.) and summer maize (*Zea mays* L.). Before the experiment, the soil properties of the 0–20 cm soil layer were as follows: organic matter (SOM) 17.3 g kg^−1^, total nitrogen (TN) 1.00 g kg^−1^, alkali-hydrolyzable nitrogen (AN) 71.33 mg kg^−1^, available phosphorus (AP) 21.6 mg kg^−1^, available potassium (AK) 108.0 mg kg^−1^, and pH 7.2.

### 2.2 Experimental design

The experiment utilized a split-plot design with six treatments. The main treatments involved rotary tillage and deep tillage during the wheat season combined with three subsidiary treatments during the maize season: no-tillage sowing, subsoiling between rows, and subsoiling within rows. The six treatments were as follows: (1) rotary tillage + no-tillage sowing (RT-NT), (2) rotary tillage + subsoiling between rows (RT-SBR), (3) rotary tillage + subsoiling within rows (RT-SIR), (4) deep tillage + no-tillage sowing (DT-NT), (5) deep tillage + subsoiling between rows (DT-SBR), and (6) deep tillage + subsoiling within rows (DT-SIR). Each treatment was replicated three times, resulting in 18 plots, each measuring 68.2 m^2^ (5.5 m × 12.4 m). Detailed treatment specifications are provided in **Supplementary Tables S1**, **S2**. During the wheat season, maize straw from the previous season was crushed twice and returned to the field. In the rotary tillage treatment, the field was tilled twice to a depth of 13–15 cm using a rotary tiller, whereas deep tillage involved plowing the field once to a depth of 28–30 cm. Subsoiling was performed at a depth of approximately 35 cm and a row spacing of 61.3 cm. Seeds and fertilizer were simultaneously added using a maize seeder, with a plant spacing of 21.9 cm. Subsoiling between rows and within rows differed in maize sowing location: the former was sown between subsoiling ditches, while the latter was sown within subsoiling ditches.

### 2.3 Soil sampling and analysis

After the wheat harvest, five soil cores were randomly collected from each plot and combined into one sample per plot. Soil samples were collected from five layers (0-50 cm) at 10 cm intervals and divided into two subsamples. The fresh portion was stored at 4°C for DOC and MBC analysis. The remaining portion was air-dried and sieved for the SOC, labile organic carbon (LOC), urease, invertase, and neutral phosphatase analysis. DOC was extracted with deionized water, centrifuged, and analyzed using a total organic carbon analyzer. MBC was determined via the chloroform fumigation–extraction method. The activities of invertase (using 3,5-dinitrosalicylic acid colorimetric method), urease (using the indophenol blue method), and neutral phosphatase (using disodium phenyl phosphate colorimetry) were measured following Guan (1986). SOC and LOC were analyzed using the oxidation with K_2_Cr_2_O_7_–H_2_SO_4_ and KMnO_4_ extraction-colorimetry (333 mmol L^−1^). Non-LOC (NLOC) was calculated as SOC minus LOC. For wheat yield measurement, a 1 m^2^ area with uniform growth was selected in each plot (3 replicates), then final yield (kg ha^-1^) was recorded and calculated after air drying and threshing.

### 2.4 Calculations

Soil carbon storage per unit volume or area is influenced by soil BD, which varied with agricultural practices. To account for BD differences, we followed the method by Ellert and Bettany (1995).


(1) $${\text{M}}_{element\;}=[\sum_{i=1}^{n}{\text{M}}_{soil,i}\times C_{i}+({\text{M}}_{j}\;-\sum_{i=1}^{n}{\text{M}}_{soil,i})\times C_{extra}]\;\times \;0.001$$

(2) $${\text{M}}_{soil,i}={\text{ρ}}_{b,i}\times {\text{T}}_{i}\times 10000$$

where M_element_ represents equivalent soil mass organic carbon storage (Mg ha^-1^); When *i*=1, 2, 3, and 4, the soil layers are 0–10, 10–20, 20–30 and 30–40 cm respectively; M_j_ represents determined equivalent soil mass. When *j*=1, 2, 3 and 4, it represents the maximum soil mass under different tillage treatments of the 0–10, 10–20, 20–30 and 30–40 cm soil layer, respectively, and the corresponding values of *n* are 1, 2, 3 and 4 respectively; M*_soil,i_* is the soil mass at each level (Mg ha^-1^); *C_i_* is the soil organic carbon content (g kg^-1^ converted to kg Mg^-1^); *C_extra_* is the organic carbon content of additional soil (kg Mg^-1^); ρ*_b,i_* is the soil BD of each layer (g cm^-3^ converted into mg m^-3^); T*_i_* is the soil layer thickness (m); coefficients 0.001 and 10,000 convert mass and area units, respectively.

The SQI was calculated by normalizing each biotic and abiotic indicator to a 0–1 scale (Jia et al., 2022):


(3) $${\text{SL}}_{\text{i}}=\frac{\text{X}}{{\text{X}}_{\text{max}}}$$

(4) $${\text{SL}}_{\text{i}}=\frac{\text{X}}{{\text{X}}_{\text{mix}}}$$

where S*_Li_* is the linear score, and X, X*_max_*, X*_min_* represent the analyzed, maximum, and minimum values of each parameter.

The overall SQI score was estimated using an SQI–area approach according to the area of a radar diagram yielded by all standard soil indicators (Kuzyakov et al., 2020):


(5) $$\text{SQI}-\text{area}=0.5\times \sum_{\text{i}}^{\text{n}}{\text{SL}}_{\text{I}}^{2\;}\times \sin\frac{2\text{π}}{\text{n}}$$

where *n* indicates the number of indicators applied for the SQI–area.

Soil enzyme activities (invertase, urease and neutral phosphatase activity) represent soil EMF. The Z-score described in Equation 5 was used to standardize enzyme activity after calculating the average to acquire a multifunctionality index.


(6) $$\text{Z}-\text{score}=(x-{\text{mean}}_{i})\;/{\text{ SD}}_{i}$$

where *x* is the enzyme activity, mean*_i_* is the average, and SD*_i_* is the standard deviation.

### 2.5 Statistical analyses

A one-way analysis of variance (ANOVA) assessed variations in soil BD, soil C components content, SOC storage, enzyme activities, SQI, EMF, and wheat yield across treatments (*p*<0.05). A *post hoc* least significant difference test compared treatments. Additionally, a three-way ANOVA was employed to investigate the main and interactive effects of year, soil depth, and tillage on various indexes. Correlation heatmap analysis explored relationships between variables. Statistical analyses were conducted using SPSS Statistics 26 (SPSS Inc, Chicago, USA), while graphs were generated using Origin Pro 2021 (OriginLabs, Massachusetts, USA). Random forest analyses were performed using the rfPermute package in R v4.0.4. Relationships between EMF and SQI were assessed via linear regression analysis with the “Im” function from the vegan package in R v.4.0.4.

## 3 Result

### 3.1 Effects of different tillage modes on soil BD

**Figure 1:**
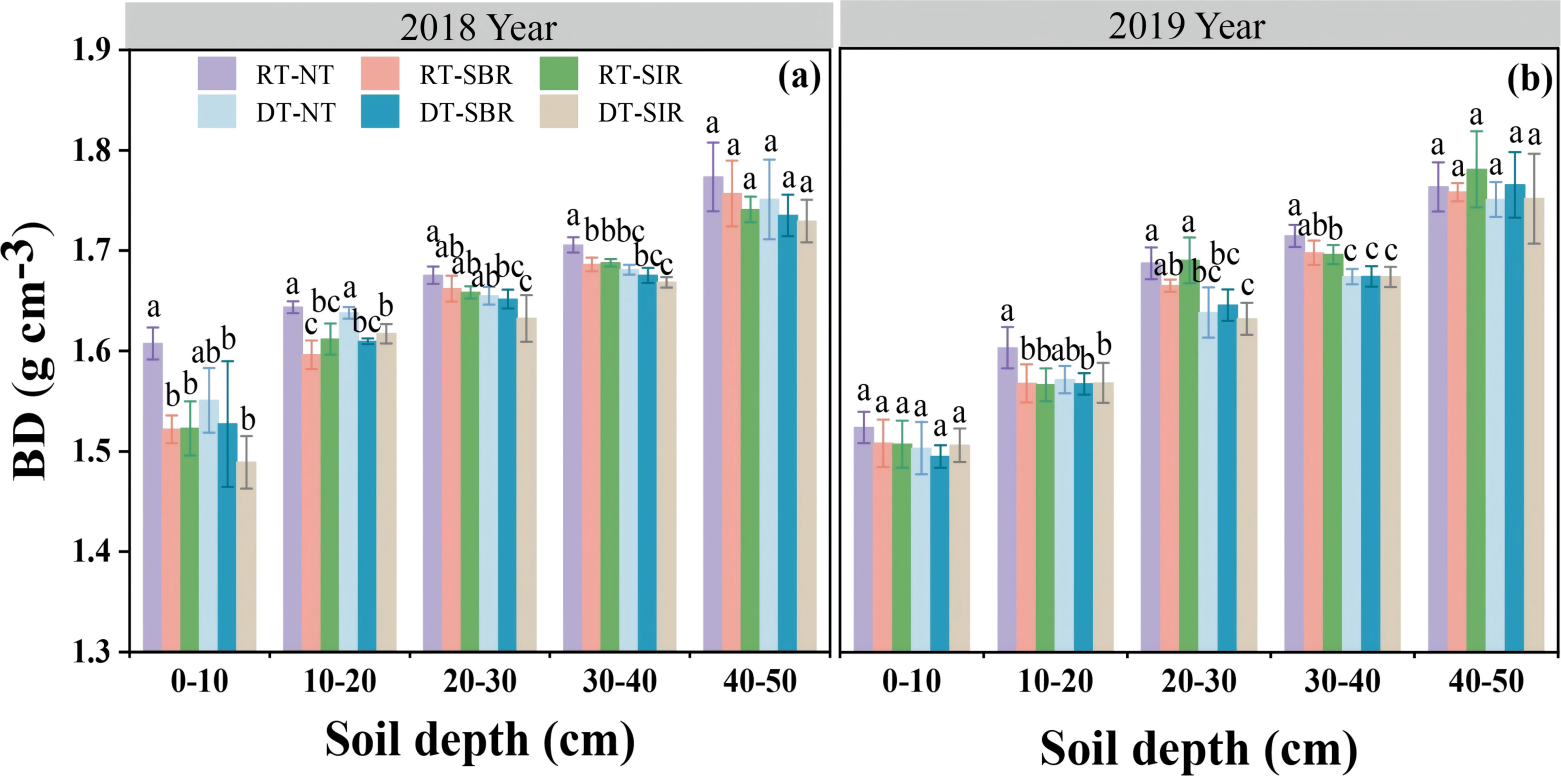
Soil bulk density (BD) in different soil layer under six treatments in 2018 **(a)** and 2019 **(b)**. Different small letters show significant differences among treatments (*p<*0.05). RT-NT, Rotary tillage + No-tillage sowing; RT-SBR, Rotary tillage + Subsoiling between rows; RT-SIR, Rotary tillage + subsoiling within rows; DT-NT, Deep tillage + No-tillage sowing; DT-SBR, Deep tillage + Subsoiling between rows; DT-SIR, Deep tillage + subsoiling within rows. The same as below.

Soil BD was affected by year (Y), soil depth (D), tillage mode (T), as well as interactions between year × soil depth and year × tillage mode (*p* < 0.05, **Table 1**). BD increased with soil depth across all treatments, while variation among treatments decreased at deeper depths. No differences in BD were observed among treatments in the 40–50 cm soil layer in 2018 and 2019 (**Figure 1**). In the 0–40 cm soil layer, BD was lower under all treatments compared to RT-NT. Furthermore, BD decreased under treatments incorporating subsoiling compared to those without subsoiling in maize in 2018, with the lowest BD of 1.49 g cm^−3^ observed under DT-SIR (**Figure 1a**). However, in 2019, no significant differences in BD were observed among deep tillage treatments in the wheat season. In the 20–40 cm soil layer, the effect of deep tillage on BD was more pronounced, while subsoiling had minimal effect on BD in 2019 (**Figure 1b**).

**Table 1:** Variance analysis under three or two factors of year, soil depth and tillage mode.

Source of variation	df	BD	SOC	LOC	NLOC	DOC	MBC	SOC storage	SC	UE	NP	SQI	EMF	Wheat yield
Year (Y)	1	13.47**	261.83**	537.71**	118.93**	540.54**	654.95**	1321.40**	157.90**	100.65**	94.34**	265.17**	0.00	265.42**
Soil depth (D)	4	657.83**	8375.91**	11925.24**	4657.67**	7216.25**	16830.59**	22940.88**	4737.60**	5648.03**	16512.65**	41889.86**	24084.80**	–
Tillage mode (T)	5	14.88**	31.39**	32.32**	21.61**	44.72**	10.44**	95.36**	23.38**	33.56**	25.77**	188.02**	50.09**	125.94**
Y × D	4	13.41**	15.11**	54.57**	26.09**	7.27**	6.66**	21.32**	26.86**	5.11**	42.45**	186.14**	8.62**	–
Y × T	5	2.66*	4.04**	1.61NS	4.98**	4.99**	6.32**	36.67**	5.32**	7.14**	8.87**	5.22**	9.32**	10.46**
D × T	20	1.28NS	10.25**	7.49**	8.89**	7.13**	26.72**	5.15**	11.72**	11.17**	16.60**	75.61**	27.79**	–
Y × D × T	20	1.12NS	5.00**	1.18NS	5.24**	1.45	6.579**	0.713	3.08**	3.01**	10.76**	3.74**	3.11**	–
Error mean squares	120	0.00	0.05	0.00	0.305	1.26	4.37	0.35	0.58	0.00	0.01	0.00	0.00	38

** Represented extremely significant test at the 1% level (*p*<0.01); * Represented significant test at the 5% level (*p*<0.05); NS represented non-significant test at the 5% level (*p*≥0.05).

BD, Bulk density; SOC, Soil organic carbon; LOC, Liable organic carbon; NLOC, Non-liable organic carbon; DOC, Dissolved organic carbon; MBC, Microbial biomass carbon; SC, Invertase activity; UE, Urease activity; NP, Neutral phosphatase activity; SQI, Soil quality index; EMF, Soil ecosystem multifunctionality.

### 3.2 Effects of different tillage modes on C components

**Figure 2:**
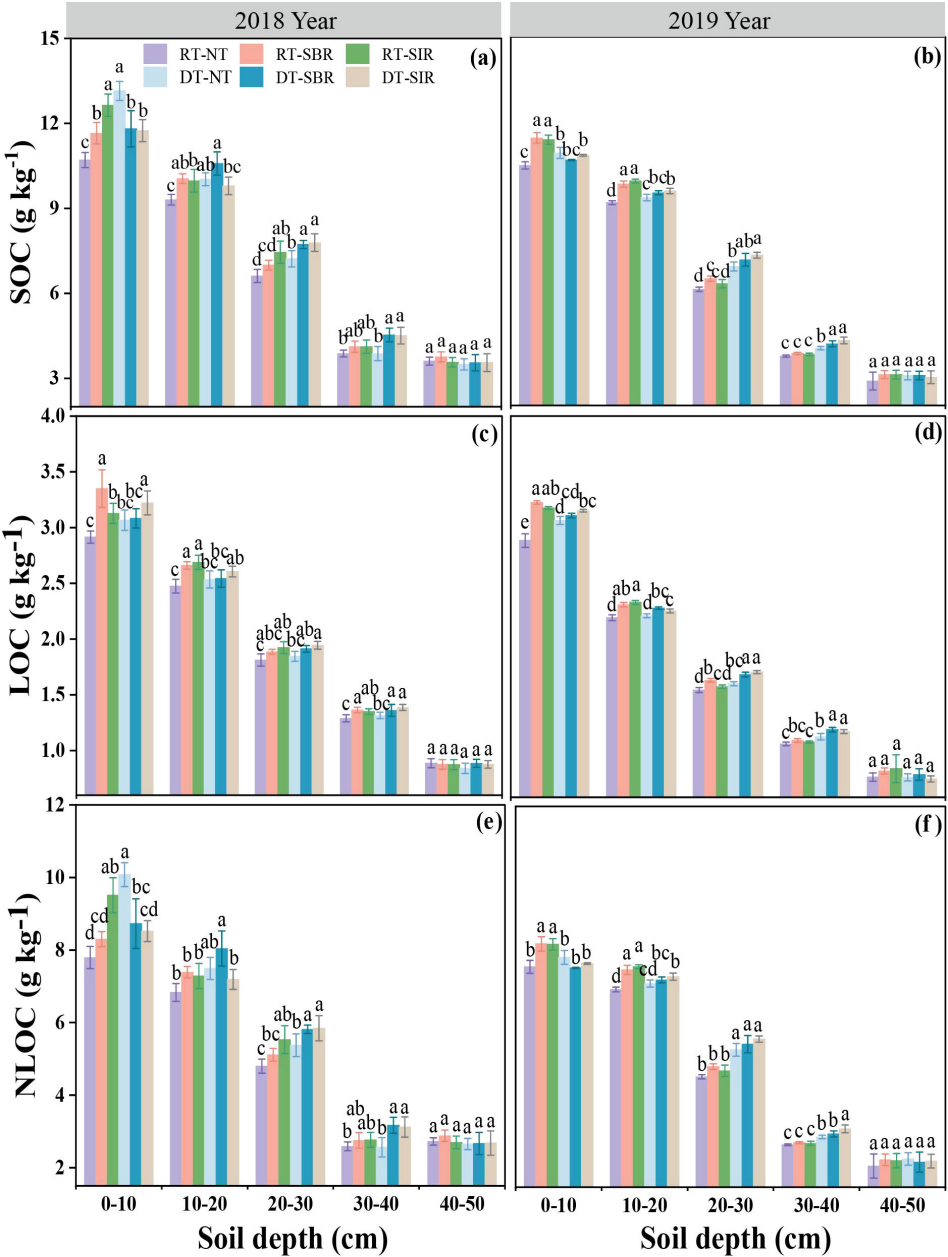
Soil organic carbon (SOC) **(a, b)**, liable organic carbon (LOC) **(c, d)** and non-liable organic carbon (NLOC) **(e, f)** in different soil layers under different treatments. Different small letters show significant difference among treatment (*p*<0.05).

SOC content was significantly affected by year, soil depth, tillage mode, and their interactions (Y × D, Y × T, D × T, Y × D×T) (*p* < 0.01, **Table 1**). SOC content consistently decreased with soil depth across all treatments (**Figures 2a, b**). Over time, the impact of deep tillage during the wheat season and subsoiling during the maize season on C accumulation has become increasingly evident. In the 0–20 cm soil layer, SOC content was higher under treatments combining rotary tillage and subsoiling than under treatments combining deep tillage and subsoiling. Conversely, in the 20–40 cm soil layer, SOC content was higher under deep tillage combined with subsoiling (DT-SIR and DT-SBR) than under treatments those with rotary tillage, with values ranging from 7.77 g kg^−1^ to 7.78 g kg^−1^ and 7.19 g kg^−1^ to 7.40 g kg^−1^ in 2018 and 2019 (**Figures 2a, b**). This indicated that deep tillage combined with subsoiling enhanced SOC accumulation in deeper soil layers.

LOC content was significantly influenced by year, soil depth, tillage mode, and the interactions between year × soil depth and soil depth × tillage mode (*p* < 0.01, **Table 1**). LOC content decreased with soil depth over the two years. In 2018, LOC content in the 0–40 cm soil layer increased under all treatments compared to RT-NT, with the highest increase being 15% (**Figure 2c**). In 2019, LOC content in the 0–20 cm soil layer reached 3.23 and 3.18 g kg^−1^, respectively (**Figure 2d**). Additionally, in the 20–40 cm soil layer, LOC content under DT-SIR and DT-SBR exceeded that under DT-NT and RT-NT in both years, peaking at 1.69 and 1.72 g kg^−1^. This indicated that deep tillage in the wheat season had a greater impact on deeper soil layers compared to rotary tillage.

NLOC content exhibited a response pattern similar to SOC content across treatments (*p* < 0.001, **Table 1**). Compared to RT-NT, NLOC content under RT-SBR and RT-SIR increased in the 0–30 cm soil layer in 2018, reaching 8.50 g kg^−1^and 10.05 g kg^−1^, respectively (**Figure 2e**). In 2019, NLOC content increased in the 0–20 cm soil layer (**Figure 2f**). Under deep tillage in the wheat season, NLOC content in the 20–40 cm soil layer also increased compared to that under DT-NT, reaching 5.81 and 5.84 g kg^−1^, respectively, in 2018 (**Figure 2e**). The effect of deep tillage persisted in 2019, while the effect of subsoiling on NLOC diminished (**Figure 2f**).

**Figure 3:**
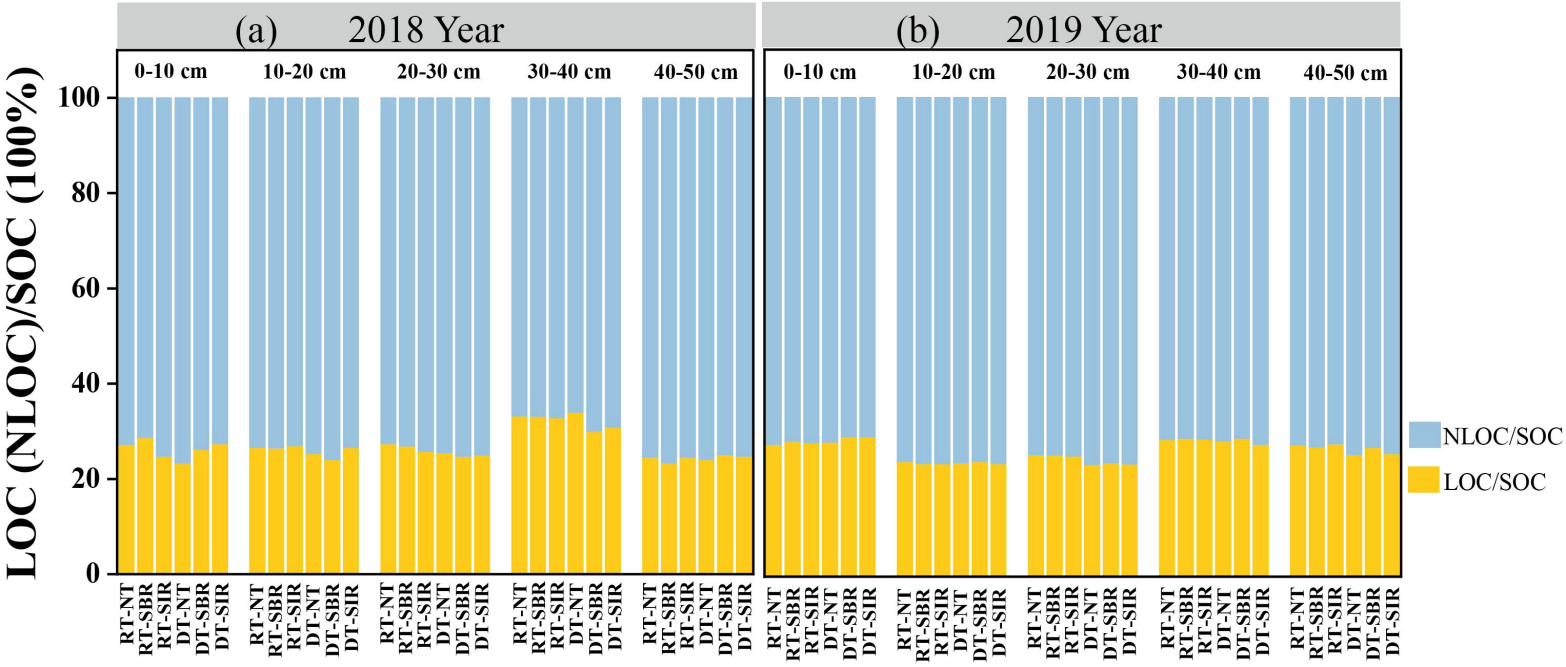
Proportion of liable organic carbon and non-liable organic carbon to total organic carbon under different treatments in 2018 **(a)** and 2019 **(b)**. Different small letters show significant differences among treatments (*p<*0.05).

NLOC accounted for the majority of the SOC, comprising 65.9%–76.6% (**Figure 3**). The LOC/SOC ratio in the 30–40 cm soil layer was higher than in other soil layers under all the treatments in both years, ranging from 30.1% to 34.1% and 27.6% to 28.9%, respectively. In 2018, the LOC/SOC ratio was higher under treatments involving rotary tillage during the wheat season in the 0–40 soil layer, although no clear trend was observed in 2019.

**Figure 4:**
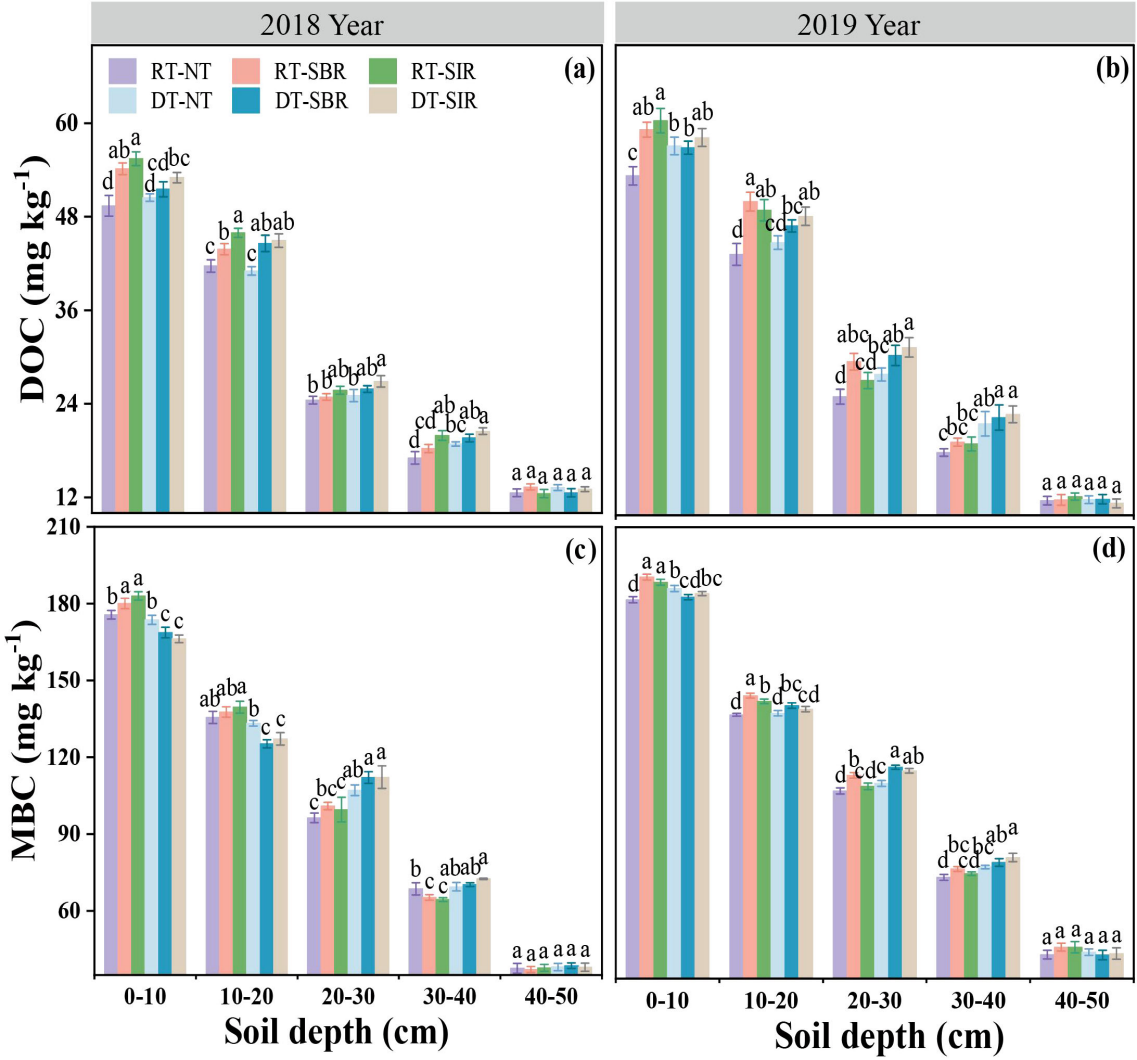
Soil dissolved organic carbon (DOC) **(a, b)** and microbial biomass carbon (MBC) **(c, d)** in different soil layers under different treatments. Different small letters show significant differences among treatments (*p<*0.05).

The DOC content was affected by year, soil depth, tillage mode, and their interactions between year × soil depth, soil depth × tillage mode, and year × tillage mode (*p* < 0.01, **Table 1**). In both 2018 and 2019, DOC content decreased with increasing soil depth. In 2018, DOC content of the 0–20 cm soil layer was higher under RT-SBR and RT-SIR compared to RT-NT, with a maximum increase of 13%. In 2019, DOC content increased in the 0–30 cm soil layer under RT-SBR and RT-SIR, reaching 59.6 and 60.7 mg kg^−1^, respectively. Under deep tillage during the wheat season, DOC content in the 0–40 cm soil layer increased in 2018 under DT-SBR and DT-SIR compared to DT-NT, reaching 51.5 and 53.0 mg kg^−1^, respectively (**Figure 4a**). Furthermore, in 2019, DOC content in the 10–30 cm soil layer increased under deep tillage combined with subsoiling treatments compared to DT-NT (**Figure 4b**).

MBC content followed a pattern similar to NLOC and SOC in response to different treatments (*p* < 0.001, **Table 1**), decreasing with soil depth in both years. In 2018, MBC content in the 0–10 cm soil layer was higher under subsoiling treatment than under no-tillage, with RT-SBR and RT-SIR peak values of 180.1 and 183.0 mg kg^−1^, respectively (**Figure 4c**). Over time, MBC content increased under RT-SBR and RT-SIR in the 0–40 cm soil layer compared to that under RT-NT, reaching 190.7 and 188.6 mg kg^−1^, respectively (**Figure 4d**). In 2018, MBC content decreased in the 0–20 cm soil layer under DT-SBR and DT-SIR compared to that under DT-NT but increased in the 10–40 cm soil layer, showing the highest increase of 5.67% over time (**Figures 4c, d**).

### 3.3 Effects of different tillage modes on SOC storage

**Figure 5:**
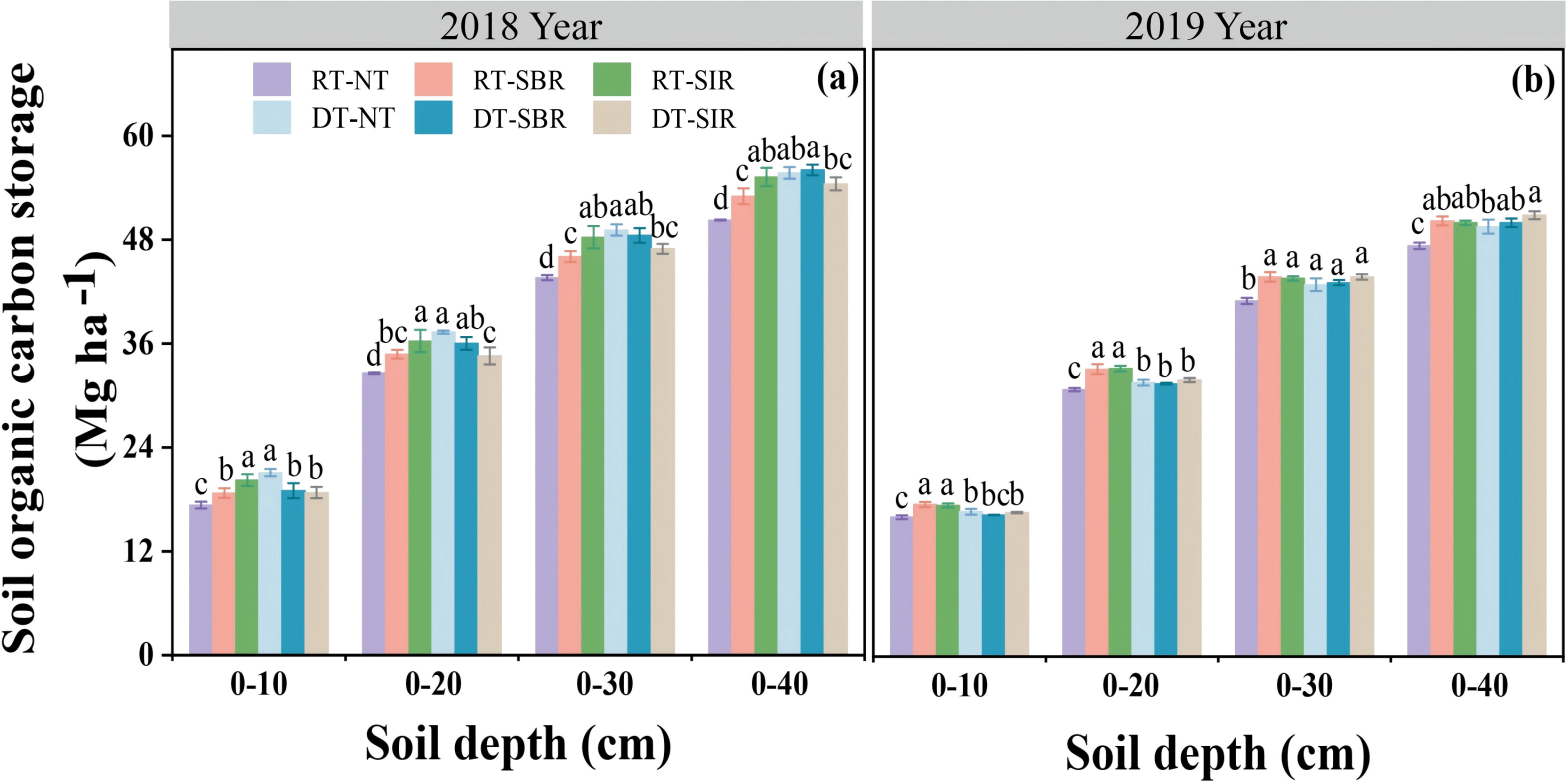
Soil organic carbon storage on equivalent soil mass basis under different tillage treatments in 2018 **(a)** and 2019 **(b)**. Different small letters show significant differences among treatments (*p<*0.05).

SOC (Equations 1, 2) storage exhibited a response pattern similar to DOC content across treatments (*p* < 0.001, **Table 1**). SOC storage increased with soil depth over two years. Compared to RT-NT, SOC storage was higher under RT-SBR and RT-SIR in all soil layers in 2018 and 2019, with values of 53.0 and 55.2 mg kg^−1^ in 2018 and 50.2 and 50.0 mg kg^−1^ in 2019, respectively (**Figure 5**). However, deep tillage combined with subsoiling decreased SOC storage compared to DT-NT in 2018 (**Figure 5a**). In 2019, no significant differences in SOC storage were observed between DT-NT and DT-SBR or DT-SIR in the 0–30 cm soil layers (*p* > 0.05, **Figure 5b**).

### 3.4 Effects of different tillage modes on soil enzyme activities

**Figure 6:**
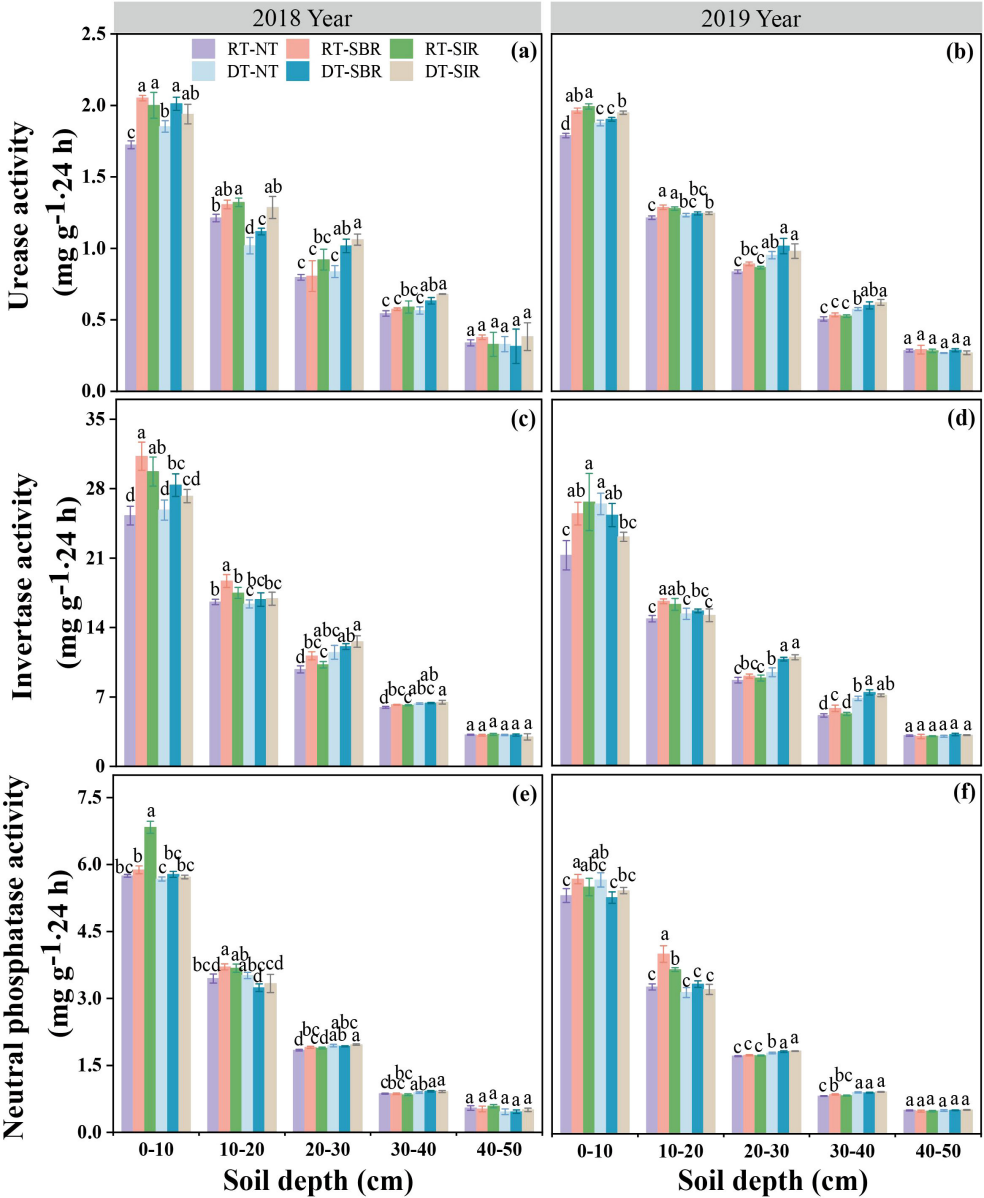
Urease **(a, b)**, invertase **(c, d)** and neutral phosphatase **(e, f)** activites in different soil layers under different treatments. Different small letters show significant differences among treatments (*p<*0.05).

The activities of urease, invertase and neutral phosphatase were was significantly affected by year, soil depth, tillage mode, and their interactions (Y × D, Y × T, D × T, Y × D×T) (*p* < 0.01, **Table 1**). Enzyme activities decreased with soil depth across all treatments in both years (**Figure 6**). Compared to RT-NT, urease activity increased under RT-SBR and RT-SIR, with the highest increases of 19.0% and 16.1%, respectively, in the 0–20 cm soil layer (**Figures 6a, b**). In 2018, urease activity was higher under subsoiling during the maize season than under no-tillage in the 0–20 cm soil layer. Urease activity also increased under DT-SBR and DT-SIR compared to DT-NT in the 0–40 cm soil layer, reaching 21.5 and 26.7 mg g^−1^·24 h, respectively (**Figure 6a**). However, in 2019, the effect of subsoiling on urease activity in the 0–50 cm soil layer was less pronounced (**Figure 6b**).

Invertase activity followed a similar trend. In 2018, invertase activity was higher under subsoiling treatment during the maize season than under no-tillage in the 0–10 cm soil layer. Compared to RT-NT, invertase activity increased under RT-SBR and RT-SIR in the 0–40 cm soil layer, reaching 31.3 and 29.7 mg g^−1^·24 h, respectively (**Figure 6c**). Compared to DT-NT, invertase activity under DT-SBR and DT-SIR maximum increased by 9.72% and 9.48% across all soil layers (**Figure 6c**). In 2019, rotary combined with subsoiling further increased invertase activity in the 0-20 cm layer. Deep tillage combined with subsoiling enhanced invertase activity in the 20–40 cm soil layer compared to no-tillage, with the highest increases of 14.1% and 16.1%, respectively (**Figure 6d**).

Phosphatase activity showed similar patterns, increasing under RT-SBR and RT-SIR in the 0–30 cm soil layer in 2018, peaking at 5.88 and 5.50 mg g^−1^·24 h, respectively (**Figure 6e**). In 2019, phosphatase activity in the 0–20 cm soil layer remained higher under RT-SBR and RT-SIR than that under RT-NT, reaching 5.68 and 5.50 mg g^−1^·24 h, respectively (**Figure 6f**). In contrast, DT-SBR and DT-SIR had no significant effect on phosphatase activity compared to DT-NT across all soil layers in 2018. However, in 2019, phosphatase activity increased under DT-SBR and DT-SIR in the 20–30 cm soil layer, reaching 1.83 and 1.85 ng g^−1^·24 h, respectively. Notably, in the 20–40 cm layer, deep tillage resulted in significantly higher phosphatase activity than rotary tillage (**Figure 6f**).

### 3.5 Effects of different tillage modes on SQI and EMF

**Figure 7:**
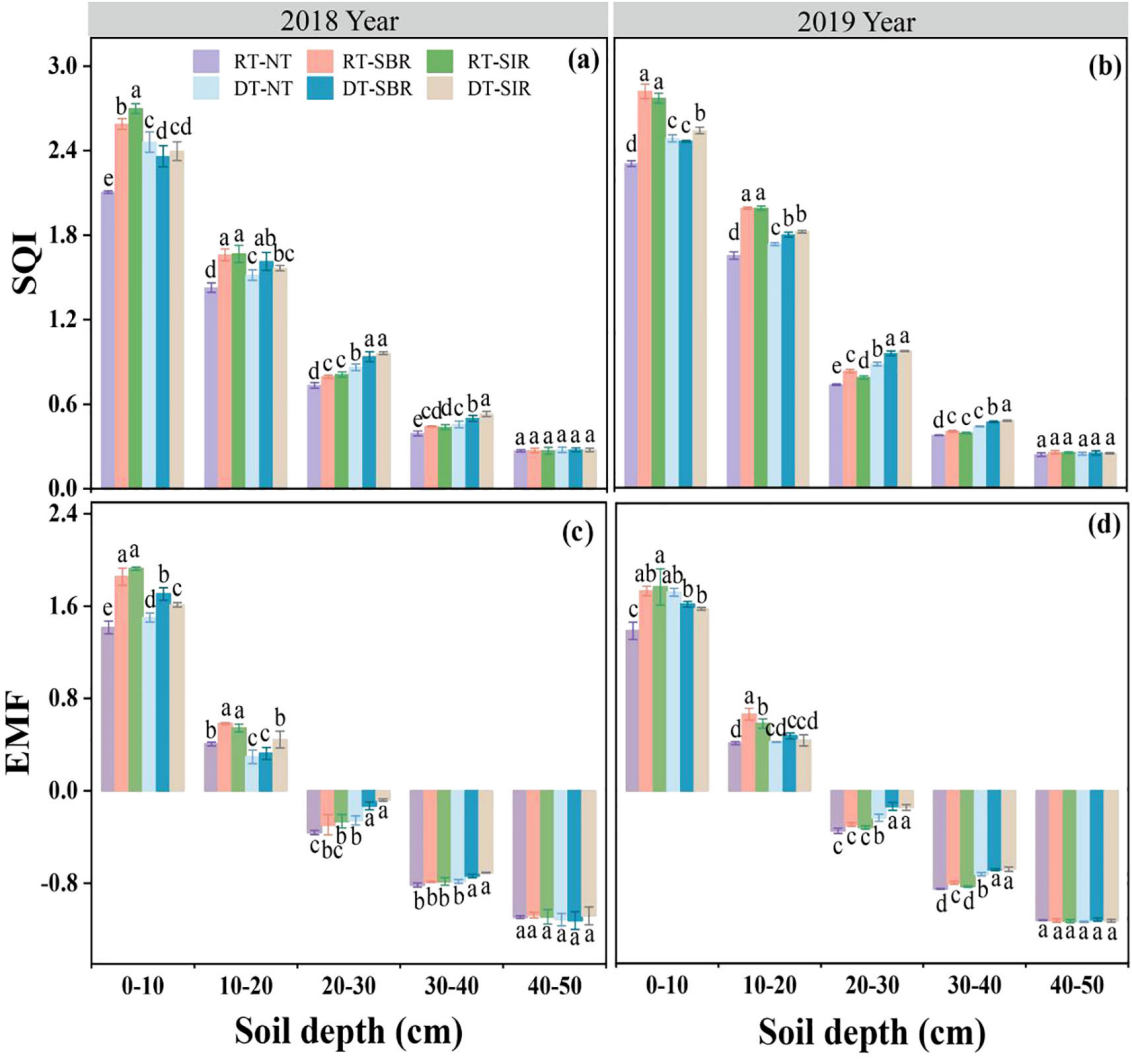
Soil quality index (SQI) **(a, b)** and ecosystem multifunctionality (EMF) **(c, d)** in different soil layers under different treatments. Different small letters show significant differences among treatments (*p<*0.05).

The response in SQI (Equations 3–5) to the different treatments had a pattern similar to enzymes activity (*p* < 0.001, **Table 1**). SQI decreased with increasing soil depth in 2018 and 2019 (**Figures 7a, b**). Compared to RT-NT, SQI increased under RT-SBR and RT-SIR in the 0–40 cm layer in both years, ranging from 0.40 to 2.81 in 2018 and 0.39 to 2.77 in 2019 (**Figure 7a**). In the 20–40 cm soil layer, SQI was higher under deep tillage than under rotary tillage in both years. Additionally, in 2018, in the 10–40 cm soil layer, SQI was higher under DT-SBR and DT-SIR compared to DT-NT, with maximum increases of 9.43% and 16.2%, respectively (**Figure 7a**). In 2019, SQI remained higher under DT-SBR and DT-SIR in the 0–40 cm soil layer compared to DT-NT, reaching value of 2.46 and 2.54, respectively (**Figure 7b**).

EMF (Equation 6) was affected by soil depth, tillage mode, and their interactions, including year × soil depth, soil depth × tillage mode, year × tillage mode, and year × soil depth × tillage mode (*p* < 0.01, **Table 1**). Similar to SQI, EMF decreased with soil depth in both years (**Figures 7c, d**). Compared to RT-NT, EMF increased under RT-SBR and RT-SIR in the 0–30 cm soil layer in 2018 and the 0–20 cm soil layer in 2019, ranging from −0.30 to 1.86 in 2018 and 0.39 to 1.93 in 2019 (**Figures 7c, d**). Furthermore, EMF was higher under RT-SBR and RT-SIR compared to DT-NT in the 0–40 cm soil layer in 2018 and in the 20–40 cm layer in 2019, ranging from –0.74 to 1.71 in 2018 and –0.71 to 1.61 in 2019. Additionally, EMF was higher under deep tillage than under rotary tillage in the 0–10 cm soil layer in 2018 and the 20–40 cm layer in 2019 (**Figures 7c, d**).

**Figure 8:**
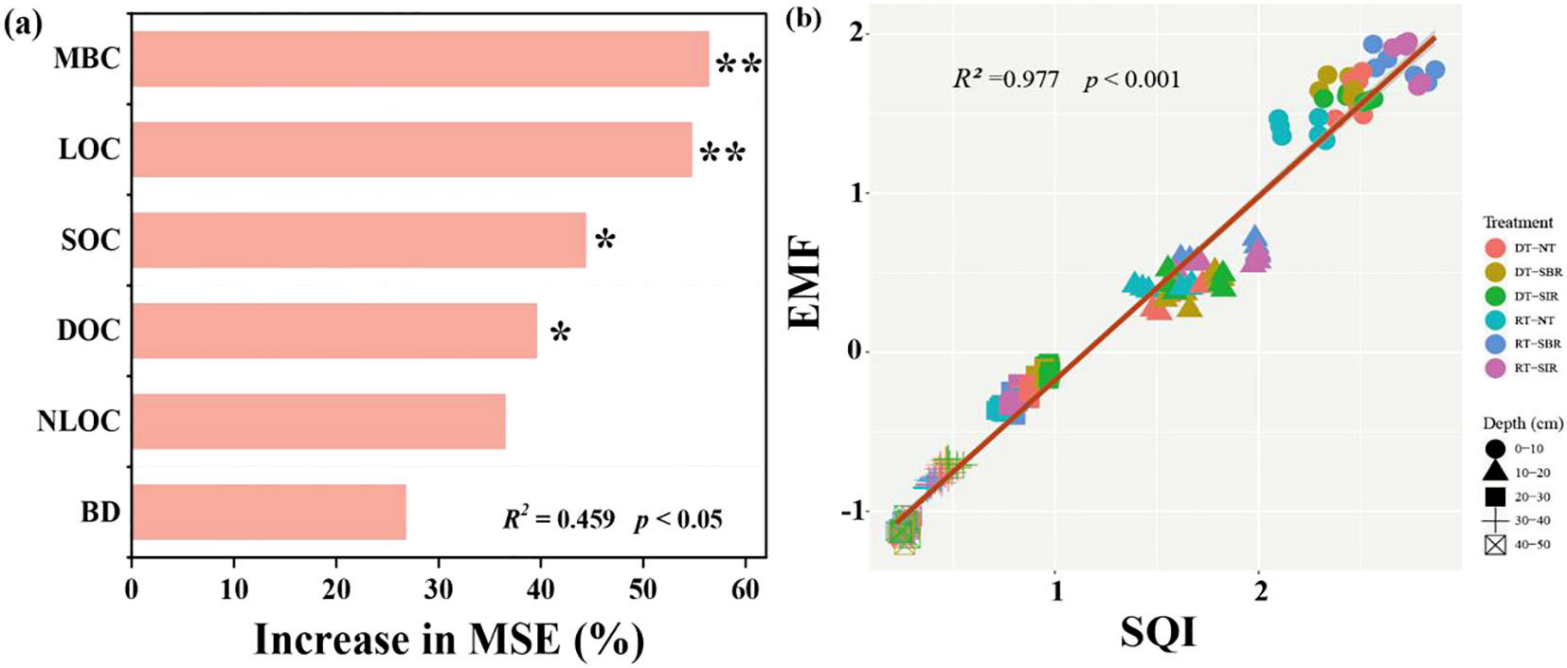
Random Forest mean predictor importance (Increase in MSE) on EMF **(a)** and relationship of soil multifunctionality (EMF) and soil quality index (SQI) by line regression analysis **(b)**. Shaded areas show 95% confidence intervals of the fit. *p <0.05; **p <0.01. MBC, Microbial biomass carbon; LOC, Liable organic carbon; SOC, Soil organic carbon; DOC, Dissolved organic carbon; NLOC, Non-liable organic carbon; BD, Bulk density.

The contributions of soil C component to EMF were estimated using the random forest model (**Figure 8a**), which explained 89% of the variation in EMF, identifying MBC, LOC, SOC, and DOC as the primary factors. EMF showed a significantly positive correlation with SQI (R^2^ = 0.977, *p* < 0.001) (**Figure 8b**).

### 3.6 Effects of different tillage modes on wheat yield

**Figure 9:**
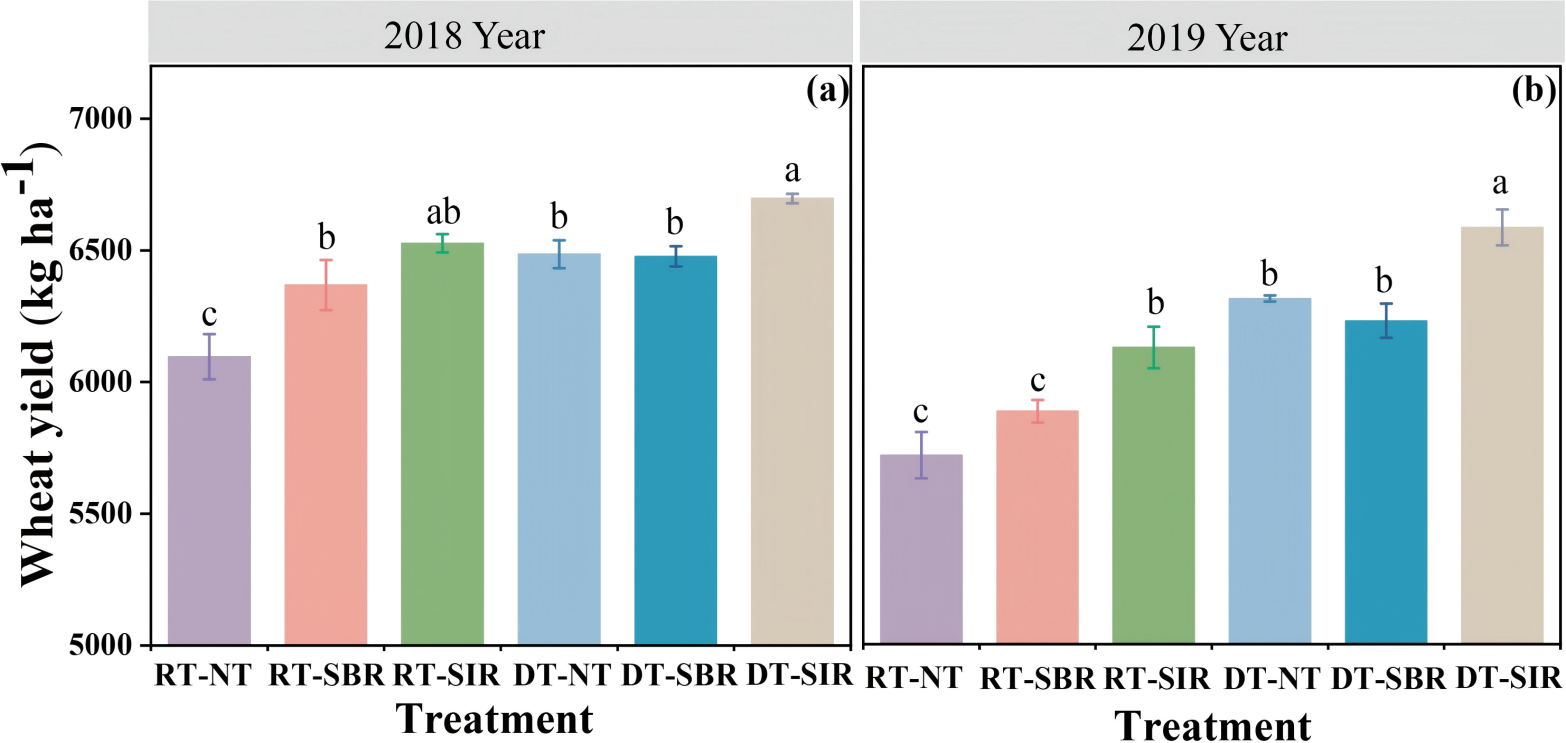
Wheat yield under different treatments in 2018 **(a)** and 2019 **(b)**. Different small letters show significant differences among treatments (*p<*0.05).

Wheat yield was strongly affected by year, tillage mode and their interactions (year × tillage mode) (*p* < 0.01, **Table 1**). Compared to RT-NT, RT-SBR and RT-SIR increased wheat yield in 2018 and 2019, with the highest values being 6527 kg ha^−1^ and 6129 kg ha^−1^, respectively (**Figure 9**). Similarly, DT-SIR enhanced wheat yield in two years compared to DT-NT, reaching 6697 kg ha^−1^ and 6587 kg ha^−1^, respectively (**Figure 9**).

### 3.7 Correlation of indexes under different tillage modes

**Figure 10:**
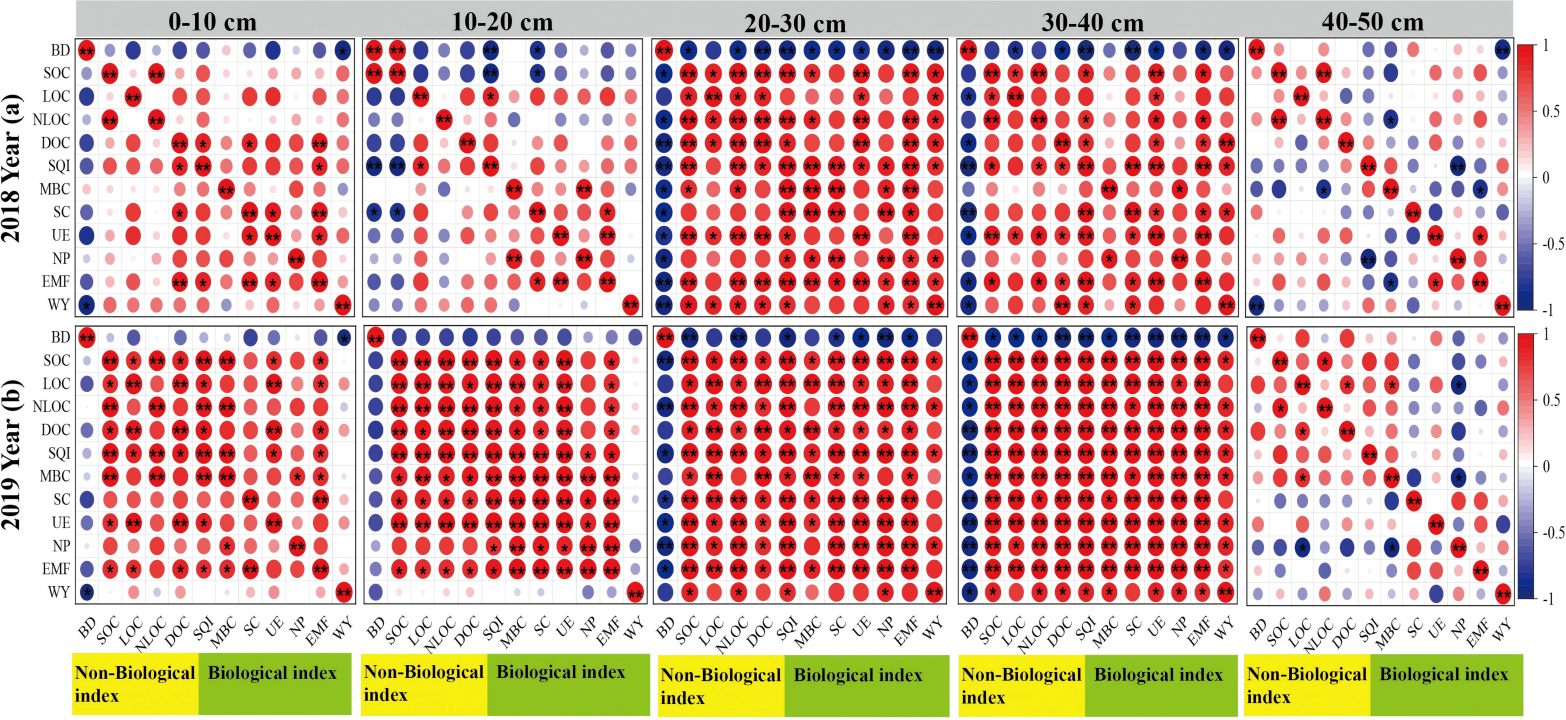
Correlation analysis of indexes in different soil layers. *p<0.05, **p<0.01; BD, Bulk density; SOC, Soil organic carbon; LOC, Liable organic carbon; NLOC, Non-liable organic carbon; DOC, Dissolved organic carbon; SQI, Soil quality index; MBC, Microbial biomass carbon; SC, Sucrase; UE, Urease; NP, Neutral phosphatase; EMF, Soil multifunctionality; WY, Wheat yield.

In both 2018 and 2019, the correlation between indicators in the 40–50 cm layer was weak (*p* > 0.05, **Figure 10**). However, in 2018, a strong correlation was observed at a depth of 20–40 cm (*p* < 0.05, **Figure 10a**). As the duration of tillage increased, correlations among various indexes in the surface soil layer became more pronounced. By 2019, all indexes showed strong correlations in the 0–40 cm soil layer (*p* < 0.05, **Figure 10b**).

## 4 Discussion

### 4.1 Effects of different tillage modes on soil BD, SOC and its storage

BD is a key physical property that significantly influences SOC content and storage (Marquez et al., 2004). Previous studies found that different tillage modes impact soil particle fragmentation through various mechanical forces, directly or indirectly affecting BD and SOC content (Abu-Hamdeh, 2003; Dam et al., 2005). For instance, Wang H. et al. (2018) found that subsoiling combined with straw return resulted in a lower BD in the 20–40 and 40–60 cm soli layer than that under moldboard plowing with crop residue removal. Subsoiling and no-tillage with straw return increased SOC content by 12.5% and 11.6%, respectively, relative to moldboard plowing, in the 0–60 cm soil layer. Conservation tillage modes exhibited higher SOC stocks compared to moldboard plowing (Wang H. et al., 2018). Conservation tillage practices, such as no tillage and subsoiling tillage, have been recommended as effective strategies for enhancing SOC accumulation (Wang et al., 2022; Zhang et al., 2023a). In our study, subsoiling during the maize season reduced BD in the 0–40 cm soil layer compared to no-tillage. Notably, BD was further decreased in the 10–40 cm soil layer under treatments involving deep tillage during the wheat season and subsoiling during the maize season in 2019, suggesting that this combination had a more pronounced and lasting effect on BD. A negative correlation was observed between BD and wheat yield, especially in the 0–10 cm and 20–40 cm layers both in 2018 and 2019 (**Figure 10**). Deep tillage during the wheat season combined with subsoiling in the maize season loosens the soil and reduces BD, benefiting crop root systems growth. Enhanced root growth, in turn, further loosens the soil, thereby reducing the BD—a result consistent with Wang H. et al. (2018). Additionally, deep tillage and subsoiling disrupt compacted tillage pan layers, increase the number of soil macropores, improve soil structure, and reduce compaction, ultimately lowering soil BD (Ma et al., 2022).

Our study also revealed that compared to RT-NT, DT-SBR and DT-SIR increased the SOC content, particularly in the 20–40 cm soil layer, aligning with the findings of Tian et al. (2016). In 2018, the LOC/SOC ratios were highest in the 30–40 cm soil layer across all treatments (**Figure 3a**). This increase may be attributed to enhanced microbial activity due to greater oxygen availability following subsoiling at a depth of 35 cm, which boosted LOC content. The DT-SBR and DT-SIR treatments exhibited the most substantial SOC storage increases in the 20–40 cm soil layer, particularly in the 20–30 cm soil layer in 2018 (**Figure 5a**). Subsoiling improved soil water retention, nutrient availability, and structural conditions, which are conducive to crop growth (He et al., 2019). This facilitated greater crop residue incorporation and carbon input. Additionally, subsoiling preserved crop residues on the soil surface, reducing soil water evaporation and maintaining soil temperature, thereby promoting humification and enhancing the C stabilization in crop residues (Ding et al., 2006; Zhang et al., 2023). Moreover, reduced soil disturbance under subsoiling minimized SOC mineralization, contributing to higher SOC stocks (Bu et al., 2020). In 2019, DT-SBR and DT-SIR treatments achieved the highest SOC storage in the 0–30 cm soil layer, with the largest increase observed in the 20–30 cm soil layer (**Figure 5b**). The deep tillage-subsoiling brought straw into deep soil layer while increasing the porosity, promoting the transformation of straw, and leading to higher SOC levels in subsoil (Wang et al., 2023; Shao et al., 2025).

### 4.2 Effects of tillage modes on organic C components

Active soil organic matter fractions, such as MBC, DOC, and LOC, are particularly sensitive to plant and microbial activities and respond quickly to agricultural management practices, making them vital indicators of soil quality (Wang W. et al., 2018; Wang et al., 2020; Jiang et al., 2022). In our study, SOC, LOC, NLOC, DOC, and MBC content decreased with increasing soil depth across all treatments. According to our previous research results (Long et al., 2019), differences in SOC content among treatments remained unchanged in the 40–50 cm soil layer between the first year of the experiment and subsequent years. However, in the 0–20 cm layer, differences among treatments began to emerge in 2018 and became more pronounced in 2019, highlighting the benefits of subsoiling in the 20–40 cm layer. In this study, rotary tillage during the wheat season combined with subsoiling in the maize season significantly increased SOC content in the 0–20 cm soil layer, consistent with the findings of Tian et al., 2016. Notably, SOC content in the 20–40 cm soil layer was higher under rotary tillage and subsoiling than under other treatments. This result suggests that deep cultivation accelerates organic matter accumulation in surface layers while redistributing nutrients in deeper layers (Krzic et al., 2003). DT-SBR and DT-SIR significantly increased DOC and MBC content in the 20–40 cm soil layer compared to other treatments. These practices reduced soil compaction, enhanced soil permeability, and improved the microecological environment, promoting aerobic microbial activity and mineral decomposition. Conversely, rotary tillage resulted in higher LOC/SOC ratios in the 20–30 cm layer, likely due to intensive soil disturbance, aggregate destruction, and thorough mixing of straw, which enhanced microbial activity and accelerated SOC mineralization (Wang et al., 2017).

### 4.3 Soil enzyme activities and EMF

Soil enzymes are pivotal to nutrient cycling and directly influence soil fertility (Nevins et al., 2021). In this study, enzyme activity varied significantly across tillage patterns and soil depths. RT-SBR and RT-SIR treatments exhibited the highest enzyme activity in the 0–20 cm soil layer, whereas DT-SBR and DT-SIR showed the highest activity in the 20–40 cm layer. Subsoiling combined with deep tillage significantly increased enzyme activity in deeper soil layers due to the incorporation of straw, promoting humus formation, enhancing soil aeration, and stimulating microbial growth (Zhu et al., 2020). However, these results differed from those of Ekenler and Tabatabai (2003). This discrepancy may be due to the incorporation of straw into the subsoiling, which disrupted compacted plow pans, improved soil structure, and encouraged root systems to release organic substances, further boosting enzyme activity in deeper layers.

As key indicators of ecosystem function, soil enzyme activities regulate multiple soil processes that occur simultaneously rather than in isolation (Wang et al., 2022). This underscores the importance of adopting integrative measures of multifunctionality. Such approaches enhance our understanding and prediction of the services soil and ecosystems provide, as well as their responses to environmental changes (Garland et al., 2021; Qiu et al., 2021). A fundamental aspect of the EMF framework is understanding nutrient cycling and the enzymes that catalyze these reactions in the soil (Nannipieri et al., 2012). Soil properties—such as nutrient availability and chemical composition—directly influence enzyme activity, which in turn impacts EMF (Nannipieri et al., 2012; Xue et al., 2020). Enhancements in soil physical properties and nutrient status further contributed to increased EMF (Ling et al., 2024; Song et al., 2024), although these improvements are often influenced by farmland management practices (Oliveira et al., 2024). For instance, Hu et al. (2024) demonstrated that applying biochar and organic fertilizer effectively enhances ecosystem functions such as crop productivity, soil nutrient storage, and enzyme activity, all of which contribute to higher EMF. In our study, we observed that both biotic and abiotic factors correlated positively with soil EMF (**Figure 10**). This indicates that carbon-related components, such as SOC, MBC, LOC and DOC, play a mediating role in influencing the EMF of agricultural soils (**Figure 8a**). Additionally, our findings revealed a significant positive relationship between EMF and SQI (**Figure 8b**), aligning with the findings of Jia et al. (2022). These correlations underscore the interconnectedness of soil properties, management practices, and ecosystem functionality.

## 5 Conclusions

**Figure 11:**
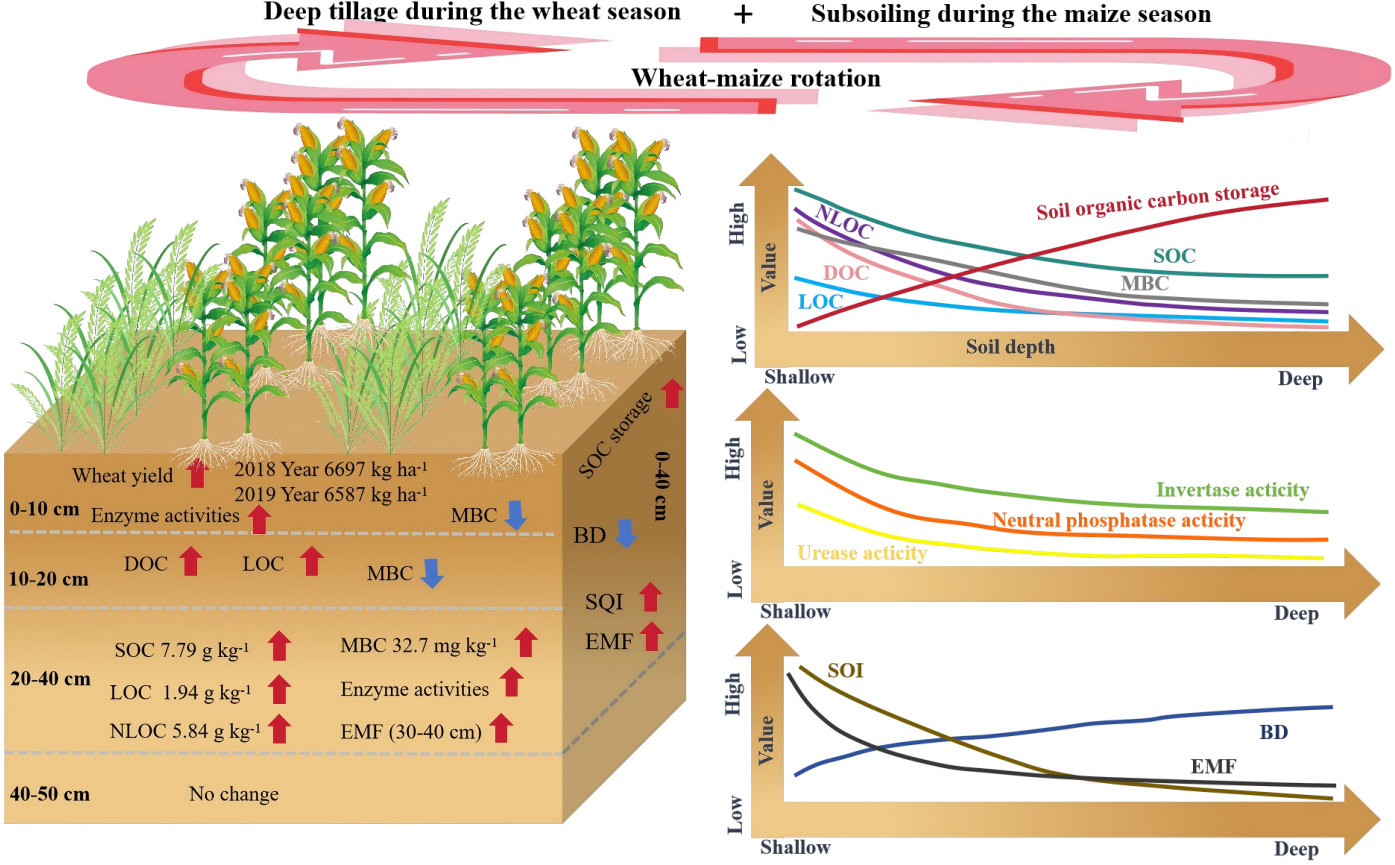
Conceptual diagram of the impact of deep tillage during the wheat season and subsoiling in the maize season on SOC content, enzyme activity, and soil ecosystem multifunctionality. The red and blue arrows indicate increase and decrease, respectively. The graph on the left represents the changes in each index for deep tillage during the wheat season and subsoiling in the maize season compared with RT-DT. The figure on the right represents the changing trend of each index with the increases in soil depth under the treatment of deep tillage during the wheat season and subsoiling during the maize season.

This study revealed that with increasing soil depth, the BD and SOC storage increased across all treatments, while C component content, enzyme activity, SQI, and EMF decreased. In addition, Subsoiling during the maize season effectively reduced BD in the 0–40 cm layer, enhanced wheat yield and increased SOC, LOC, NLOC, and MBC content in the 20–40 cm layer. Combined rotary tillage during the wheat season and subsoiling in the maize season improved SOC storage, enzyme activities, SQI, and EMF, promoting carbon sequestration, especially in deeper soil layers (**Figure 11**). This highlights the benefits of subsoiling as an effective agricultural practice for enhancing soil quality and sustainability.

## Data Availability

The original contributions presented in the study are included in the article/**Supplementary Material**. Further inquiries can be directed to the corresponding authors.
