# Supplementary material for: Soil organic carbon and ecosystem multifunctionality are enhanced by subsoiling in fluvo-aquic soil of North China Plain
Source: Front Plant Sci. 2025 Apr 16;16:1559653. doi: 10.3389/fpls.2025.1559653 (PMC12040894; doi:10.3389/fpls.2025.1559653)
Supplement: Supplementary file 1 [file Table1.docx]

Table S1 The detailed tillage information under each treatment

| Treatment | Tillage in wheat season | Tillage in maize season |
| --- | --- | --- |
| RT-NT | Rotary tillage | All straw was returned to the field after mechanical harvesting of wheat.  The maize-no-tillage seeder was used for simultaneous sowing of seed and fertilizer (sowing maize next to crop stubble). |
| RT-SBR |  | All wheat straw was returned to the field after mechanical harvest. The subsoiler was used for subsoiling in the field. Seed and fertilizer were sown simultaneously with a maize seeder, and maize was sown between subsoiling furrows. |
| RT-SIR |  | All straw was returned to the field after mechanical harvesting of wheat. The subsoiler was used for subsoiling in the field. Seed and fertilizer were sown simultaneously with a maize seeder, and maize was sown in subsoiling furrows. |
| DT-NT | Deep tillage | All straw was returned to the field after mechanical harvesting of wheat.  The maize-no-tillage seeder was used for simultaneous sowing of seed and fertilizer (sowing maize next to crop stubble). |
| DT-SBR |  | All wheat straw was returned to the field after mechanical harvest. The subsoiler was used for subsoiling in the field. Seed and fertilizer were sown simultaneously with a maize seeder, and maize was sown between subsoiling furrows. |
| DT-SIR |  | All straw was returned to the field after mechanical harvesting of wheat. The subsoiler was used for subsoiling in the field. Seed and fertilizer were sown simultaneously with a maize seeder, and maize was sown in subsoiling furrows. |

Table S2 Varieties of wheat and maize and fertilization

| Variety | Base fertilizer (kg ha^-1^) | topdressing（urea）(kg ha^-1^) |
| --- | --- | --- |
| Zhengmai 369 | 750（N: P_2_O_5_: K_2_O=20:16:16） | 150（N 69） |
| Jundan 29 | 750（N: P_2_O_5_: K_2_O=28:10:12） | 0 |
